# Natural Killer Cells and Dendritic Cells: Expanding Clinical Relevance in the Non-Small Cell Lung Cancer (NSCLC) Tumor Microenvironment

**DOI:** 10.3390/cancers13164037

**Published:** 2021-08-11

**Authors:** Pankaj Ahluwalia, Meenakshi Ahluwalia, Ashis K. Mondal, Nikhil S. Sahajpal, Vamsi Kota, Mumtaz V. Rojiani, Ravindra Kolhe

**Affiliations:** 1 Department of Pathology, Medical College of Georgia, Augusta University, Augusta, GA 30912, USA; pahluwalia@augusta.edu (P.A.); amondal@augusta.edu (A.K.M.); nsahajpal@augusta.edu (N.S.S.); 2 Department of Neurosurgery, Medical College of Georgia, Augusta University, Augusta, GA 30912, USA; mahluwalia@augusta.edu; 3 Department of Medicine, Medical College of Georgia, Augusta University, Augusta, GA 30912, USA; vkota@augusta.edu; 4 Department of Pharmacology, Penn State University College of Medicine, Hershey, PA 17033, USA; mrojiani@pennstatehealth.psu.edu

**Keywords:** NK cells, natural killer cells, DCs, dendritic cells, immunotherapy, prognostic, predictive, cancer, gene expression, lung cancer, therapeutics, NSCLC

## Abstract

**Simple Summary:**

Cancer is one of the leading causes of mortality around the globe. In the past decades, there has been rapid progress in the development of tools to detect, screen, and treat several cancers. For its benefit to reach a wider patient population, significant challenges such as tumor heterogeneity, resistance to therapies, and lack of biomarkers should be addressed. The immune system holds the key to a greater understanding of these complex barriers. Natural Killer cells are cytotoxic cells of innate immunity that can kill multiple tumorigenic cells. Dendritic cells link innate and adaptive immunity by processing and presenting tumor-derived antigens to initiate anti-tumor T cell response. These immune cells and associated gene signatures have emerged as potential biomarkers with prognostic and predictive potential in several cancers. In this review article, we have discussed the biological roles of NK cells and DCs along with their translational relevance in NSCLC.

**Abstract:**

Non-small cell lung cancer (NSCLC) is a major subtype of lung cancer that accounts for almost 85% of lung cancer cases worldwide. Although recent advances in chemotherapy, radiotherapy, and immunotherapy have helped in the clinical management of these patients, the survival rate in advanced stages remains dismal. Furthermore, there is a critical lack of accurate prognostic and stratification markers for emerging immunotherapies. To harness immune response modalities for therapeutic benefits, a detailed understanding of the immune cells in the complex tumor microenvironment (TME) is required. Among the diverse immune cells, natural killer (NK cells) and dendritic cells (DCs) have generated tremendous interest in the scientific community. NK cells play a critical role in tumor immunosurveillance by directly killing malignant cells. DCs link innate and adaptive immune systems by cross-presenting the antigens to T cells. The presence of an immunosuppressive milieu in tumors can lead to inactivation and poor functioning of NK cells and DCs, which results in an adverse outcome for many cancer patients, including those with NSCLC. Recently, clinical intervention using modified NK cells and DCs have shown encouraging response in advanced NSCLC patients. Herein, we will discuss prognostic and predictive aspects of NK cells and DC cells with an emphasis on NSCLC. Additionally, the discussion will extend to potential strategies that seek to enhance the anti-tumor functionality of NK cells and DCs.

## 1. Introduction

Lung carcinoma is one of the deadliest cancers globally, with almost 1.8 million deaths annually [1]. Lung cancer consists of heterogenous subtypes that can be differentiated at epidemiological, histological, and molecular levels. Small-cell lung cancer (SCLC) accounts for 13%, while non-small cell lung cancer (NSCLC) causes 76% of lung cancer cases [2]. Tobacco smoking has a well-documented association, with a high prevalence of lung carcinoma. Furthermore, air pollution (household and outdoor) is the second most leading risk factor of lung cancer [3]. According to one estimate, more than 91% of the global population lives under WHO air quality guideline levels [4]. Air pollution consists of fine particulate matter (PM_10_ and PM_2_._5_), gases (oxides of sulfur and carbon), and metals (lead, nickel, and vanadium). According to one meta-analysis, an increase in every 10 μg/m^3^ of PM_10_ levels leads to a 22% higher risk of lung cancer [5]. The incidence and mortality of lung cancer are falling in the United States due to strengthened tobacco control and public education, but are rising in developing countries [6]. In the United States, from 2008 to 2016, NSCLC related incidence decreased at an annual rate of 3.1%. The specific survival in lung cancer patients improved from 26% in 2001 to 35% in 2014 [2]. The advances in treatment regimens including the incorporation of targeted therapies and immunotherapies are major factors in this improvement. Surgical resection of the tumor is most effective for stage I, stage II, and some stage III NSCLC cases [7]. Unfortunately, most of the patients have tumor recurrence with a 5-year survival rate that falls from 83% in stage IA to 36% for stage IIIA patients [8]. Platinum compounds have been used against cancer since the 1978 approval by the FDA [9]. Platinum drugs cause damage to tumor cells by cross-linking DNA. In healthy cells, efficient DNA damage response can repair the damage, but due to changes accumulated through the process of cancer evolution, most of the cancer cells lack DNA repair pathways and are subsequently killed [10]. Platinum-based doublet therapies (cisplatin combined with docetaxel or other cytotoxic compounds) have been standard of care for advanced NSCLC patients with a good performance status [11]. Besides these, platinum triplets (addition of a third component—bevacizumab, an anti-VEGF moAb) are recommended for advanced metastatic NSCLC [12,13]. For NSCLC patients with activating EGFR mutations (30–50% of Asians and 15% Caucasians), tyrosine kinase inhibitors (TKIs) comprise the first-line therapeutic strategy [14]. Encouragingly, recent breakthroughs in immunotherapies have provided a new direction to the management of lung cancer patients. Currently, patients with higher expression of PD-L1 are administered pembrolizumab (anti-PD-1) as a first- and second-line therapy in patients with advanced NSCLC [15]. Atezolizumab (anti-PD-L1) and Nivolumab (anti-PD-1) are approved as a second-line therapy, while Durvalumab (anti-PD-L1) is used as a maintenance therapy in unresectable stage III NSCLC patients [15,16,17].

**Figure 1:**
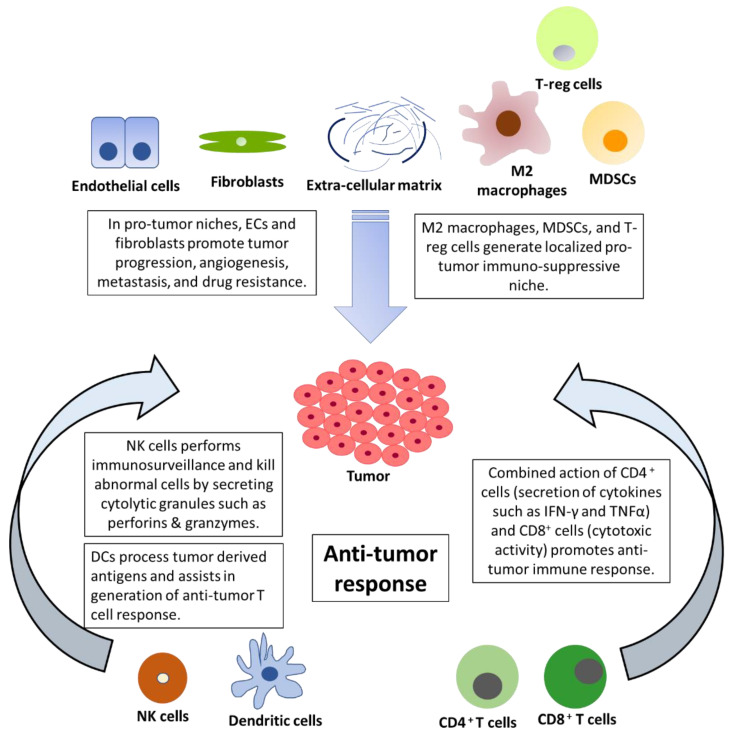
The opposing forces in the tumor microenvironment: the pro-tumor roles of fibroblasts, ECM, and suppressive immune cells against the anti-tumor immune response of NK cells, DCs, CD4^+^, and CD8^+^ T cells.

The tumor microenvironment is composed of malignant cells along with fibroblasts, extracellular matrix (ECM), endothelial cells, adipocytes, and immune cells [18] (Figure 1). Tumors can be divided into two major immunological subtypes: hot (high T cell activity) and cold (lack of T cell priming/activation) based on inflammatory cytokine profile [19]. In the cancer progression cycle, excessive secretion of PD-L1 in the tumor and enhanced PD-1 signaling inactivates T cells leading to tumor growth and metastasis [20]. The use of novel immunotherapeutic agents, such as nivolumab (anti-PD-1), pembrolizumab (anti-PD-1), and atezolizumab (anti PD-L1), has led to the improved overall survival of NSCLC patients compared to treatment with docetaxel [16,21,22]. Further, the combination of cytotoxic therapy and immunotherapy has shown synergistic improvements in the anti-tumor response. The combination of pemetrexed (folate antimetabolites), carboplatin (platinum-based antineoplastic drug), and pembrolizumab (anti-PD-1) led to the improvement of the objective response rate and progression-free survival [23,24]. This striking difference was exhibited in the benefits of immunotherapy, as hot tumors show a higher response rate to immunotherapy. The immune checkpoint blockade (ICB) is effective in patients with expression of PD-L1 or high TMB (tumor mutation burden), but there are different approaches required to target cold tumors. TMB is the number of nonsynonymous coding mutations per million bases (Mb) and is a predictive biomarker for ICB therapy efficacy. It is associated with increased secretion of neoantigens and cytotoxic T cell activity in several cancers [25,26]. There is increased interest in exploring strategies to convert cold tumors to hot tumors in an attempt to increase immunotherapy benefits [27]. Recent breakthroughs in the form of personalized RNA mutanome vaccine based on individuals’ genomic information activates lymph-node-DCs and can generate potent T cell response [28]. The ICBs have remarkably improved survival but their benefits are achieved only in a minority of patients. Furthermore, the defects in antigen presentation in the form of reduced expression of HLA class I or loss of *B2M* can cause resistance to checkpoint inhibitors [29]. Additionally, IFN-γ signaling and corresponding higher production of IDO1 (Indoleamine 2, 3-dioxygenase 1) can also lead to poor response to checkpoint inhibitors [29,30,31]. The IDO expression is promoted by pro-inflammatory stimuli generated by IFN-γ and might result in a suboptimal anti-tumor immune response in cancer patients. Furthermore, APCs with IDO production can activate and promote the production of immunosuppressive regulatory T cells [31]. Despite several therapeutic challenges at the tumor level, recent advancements in the form of adoptive T-cell therapy have shown a durable response in cancer patients. There are several difficulties in its clinical application due to tumor heterogeneity, fewer neoantigens, systemic cytokine toxicities, and challenges associated with the production of cells in compliance with GMP (Good Manufacturing Practice) [32,33]. Recently, interest in NK cells has emerged as a promising alternative due to their killing abilities and as a safer alternative to adoptive T cell therapy because of its lower immune-related adverse events [34]. In this review article, we have discussed the therapeutic and prognostic benefits of NK cells and DCs with a focus on NSCLC.

## 2. The Emerging Role of NK Cells in the Tumor Microenvironment and Immunotherapy

Natural killer cells are part of the innate lymphoid family and represent up to 5–20% of circulating lymphocytes [35]. Innate lymphoid cells (ILCs) are non-B and non-T lymphocytes that lack antigen specificity and provide immediate protection against pathogen and cancer [36]. These cells are grouped into three broad categories based on their expression of transcription factors and cytokine profile. Type 1 ILCs correspond to IFN-γ-producing NK cells and ILC1s. ILC2s secrete T_h_2 (T helper cells) cytokines and ILC3s pre-dominantly secrete T_h_17(T helper 17 cells) cytokines [36]. Conventional NK (NK) cells undergo development in the bone marrow and are present in circulation. Tissue-resident NK cells (trNK) have been found in mucosal tissues, such as tonsil, gut, skin, lung, and endometrium, along with non-mucosal tissues, such as liver, bone, and spleen [37,38]. NK cells are killer cells and can identify and eliminate stressed cells that may be dangerous to the host particularly during viral infection or cancer [39]. Upon identification of cancer cells, NK cells form immune synapses and secrete cytolytic molecules, such as perforins and granzymes, to kill the abnormal cells [40]. Due to the diverse, organ-specific chemokine profile, NK cells showcase a phenotypically plastic population [41]. NK cells are predominantly defined as CD3–CD56+ but the expression of markers may be altered in different tissues. They show an absence of TCR and CD3 molecule but highly express CD56, also called neural cell adhesion molecule (NCAM). Cytokine producing NK cells in secondary lymphoid organs such as lymph nodes are CD56^bright^CD16^lo^, whereas highly toxic NK cells in circulation are CD56^dim^CD16^hi^ [42,43]. It was believed earlier that the cytotoxic effects of NK cells are part of the innate immune response and are, therefore, short-lived, but recent observations have pointed out the presence of ‘immune memory’, whereby these cells show higher functional activity and can generate heightened responses similar to T and B cells [44,45]. In a recent study, human NK cells exhibited memory recall response against viral antigens [46]. In another study, clonally expanded adaptive NK cells generated a response that resembled an adaptive response against human cytomegalovirus (HCMV) [47].

The process of development and maturation of NK cells makes them competent to identify and kill host cells with aberrant expression of MHC class I molecules (called human leukocyte antigen—HLA) in humans [48]. During NK cell maturation, the interaction between inhibitory killer cell immunoglobulin-like receptors (KIRs) and HLA provides functional competency to NK cells. This process termed ‘licensing’ suppresses NK cell function in the presence of intact MHC and minimizes the destruction of healthy cells [49]. This suppression is eliminated in the presence of downregulated or altered MHC expression as in tumor cells [50]. Aberrant cells can also be killed by antibody-dependent cell cytotoxicity (ADCC), whereby NK cells bind to the Fc region of a tumor cell-bound antibody [51]. The mechanism of killing by NK cells lies in part in their appearance as ‘Large granular lymphocytes’ [52]. When a susceptible cell is identified by the NK cells, specific lytic granules converge toward the immunological synapse through microtubules [53]. There are two major components in these lytic granules—perforins and granzymes. Perforins are cytolytic proteins that are inserted into the plasma membrane and leads to osmotic lysis in a Ca^2+^-dependent manner [50,54]. Perforins are found to play a critical role in controlling tumor metastasis [55,56]. Granzymes are serine proteases that activate caspase signalling and leads to apoptosis of the target cell [57]. Natural killer cells can kill more than a single cell through their degranulation process. NK cells form multiple contacts with target cells and can sequentially kill several abnormal cells in a time-dependent manner [58]. NK cells express a high mRNA pool of granzymes and perforins that are rapidly translated to protein when required [59]. Interestingly, upon a single encounter, an NK cell releases only one-tenth of its cytotoxic lytic granules but it has been determined that even a single granule is sufficient to induce target cell death [54]. NK cells have been shown to shift from fast GrzB-mediated cell death to slow death receptor-mediation killing in the later stages and can serially kill up to 30+ tumor cells [60].

NK cells play an important role in tumor immunosurveillance, which is emphasized by the association between NK cell deficiency and cancer [61,62]. In a large prospective study with 11 years of follow-up period, patients with medium or high NK cell cytotoxic activity were found to be associated with a lower risk of cancer. In this study, NK effector cells isolated from peripheral blood were used to measure specific lysis of target cells (K562, leukemia cell line) using the ^51^Cr-release assay [63]. In a recent meta-analysis, infiltration of NK cells was found to be associated with better overall survival in solid tumors [64]. NK cells express heparinase to invade primary tumors, and therefore, affect tumor progression [65]. Furthermore, NK cells can also impact metastasis by eliminating circulating tumor cells [39]. There are several ways through which the function of NK cells is negatively affected in an immune-suppressive milieu of the immune-evasive tumor. The enriched metabolites of the kynurenine pathway contribute to the immune escape of cancer cells [66]. This escape is fueled by the secretion of kynurenine by cancer cells, which subsequently leads to immune tolerance in the tumor microenvironment, induction of immunosuppressive T-regulatory cells, and blockade of IL-2 [66]. In the presence of TGF-β, the gene expression profile of NK cells shifts toward lower cytotoxicity. Activin-A binds to type I activin receptor ALK4, which is present on NK cells and suppresses their metabolism [67]. Furthermore, at the metabolic level, impaired glycolysis in NK cells due to overexpression of fructose 1,6-bisphosphatase impairs NK cell activity [68]. In this study, NK cells were shown to prevent tumor initiation in lung cancer but failed to prevent tumor progression due to the metabolic dysfunctional state of NK cells [68]. Further, low levels of nutrients such as glucose and a hypoxic environment suppress the anti-tumor activity of NK cells [69]. Additionally, in the TME, a high concentration of lactate and low pH can also impair NK cells [69,70].

In many tumors, there is poor infiltration of NK cells. Adoptive transfer of NK cells with ‘memory-like status’ was induced when exposed to a combination of cytokines such as IL-12, IL-15, and IL-18, and this has been proposed to enhance tumor immunity [71,72]. Ex vivo activation and genetic modification of NK cells can greatly increase anti-tumor immunity while overcoming resistance [73]. In another study, blocking of NKG2A, an inhibitor of NK activation, led to improved survival in preclinical solid tumor models. In this study, a combination of peptide vaccination and NKG2A-blocking antibodies led to improved CD8+ T Cell Immunity [74]. In another approach, IL-15 capacity to stimulate NK cells has yielded clinical benefits. In NSCLC, a subcutaneous IL-15 super-agonist named ALT-803, showed NK cell expansion and strong local inflammation in patients with advanced cancer [75]. In another trial of metastatic NSCLC patients, ALT-803 addition to nivolumab improved clinical response in patients with PD-1 relapsed or refractory disease [76]. IL-15 super-agonists and their role in the expansion of NK cells offers new approaches, which can be combined with existing treatment regimens with a promise of increased anti-tumor response. ICB therapies require neo-antigen presentation through MHC-I and the activation of CD8+ T cells. On the other hand, NK cells respond to oncogenic stress through germline-encoded receptors, such as activating receptors (NKG2D), natural killer receptors (NKp30), and Killer cell immunoglobulin-like receptors (KIRs) [77]. The balance of inputs through the expression level of NK cell ligands and tumor ligands dictates the outcome of NK-tumor cell interaction. The interaction favors lysis in the presence of cytokines, such as IL-15, IL-21, IL-18, and IL-12, and stimulatory NK receptors NKG2D, DNAM-1, NKp30, NKp44, NKp46, and NKG2C, among others. NK cells prevent lysis in the presence of suppressive factors, such as TGF-β, ACVR1, and A2AR, and inhibitory NK receptors, such as PD1, TIGIT, and NKG2A [78,79].

NK cells also play a critical role in mediating adaptive immune response. Activated NK cells express a variety of cytokines, including IL-5, IL-10, IL-13, G-CSF, GM-CSF, M-CSF, TNF-alpha, IFN-γ, and others [80,81]. IFN-γ, particularly, can play a potent role in activating other cells, especially macrophages, and dendritic cells. IFN-γ induces heightened inflammatory cytokine production, phagocytic capacity, enhanced MHC expression, and activation of adaptive immunity [82,83]. IFN-γ also plays an anti-tumorigenic effect by enhancing antigen processing and presentation machinery in tumor cells [84]. Interestingly, IFN-γ is essential for the maturation of dendritic cells as it upregulates the expression of MHC (MHC-I and MHC-II) and co-stimulatory molecules essential for T cell activation [84]. Furthermore, IFN-γ induces the expression of IL-12 and IL-15 in DCs, which induces anti-tumor responses through CD4^+^ T_H_1 and CD8^+^ T cells [85,86].

NK cells act as a key regulator of DC recruitment and retention in the tumor microenvironment. NK cells recruit cDC1 into the tumor microenvironment through the secretion of chemokine CCL5 and XCL1. It was found that the expression of these chemokines along with NK and cDC1 was associated with improved overall survival. Interestingly, this study showed that recruitment of DCs was sensitive to immune-suppressive Prostaglandin E2 (PGE2) [87]. Further NK cells also produce cytokine Fms-related tyrosine kinase 3 ligand (*FLT3LG*). *FLT3LG* was found to be positively associated with an increase in or a majority of DCs [88]. NK cells regulate the infiltration of DCs in the TME and subsequently affect T cell priming, thus, playing a critical anti-tumor role.

NK cells are present in most tissues, but their distribution is tissue-specific, ranging from lower to higher cytotoxic properties [89]. Increased infiltration and functionality of NK cells ha been associated with increased overall survival in several cancers, including NSCLC [90]. Chemokine signaling plays an important role in regulating the influx of NK cells in the TME. In NSCLC, higher infiltration of CD56^bright^ NK cells was found to be linked with downregulation of CXCL2 and upregulation of CXCL9, CXCL10, and CCL19 [89]. Furthermore, increased expression of chemokine C-X3-C motif ligand 1 (CX3CL1), which is a known ligand for the CX3CR1 receptor present on NK cells, was found to be associated with improved overall survival of patients with lung cancer [91]. Even in the presence of proper chemokine signaling, NK cells may fail to enter the tumor tissue. In NSCLC tissues, it was found that NK cells were found predominantly present in the stroma with cytokine secreting activity and not in contact with tumor cells [92]. Therefore, the chemokine profile of the tumor tissue and stromal barriersneeds to be dissected further for a complete understanding of the interaction between tumor and NK cells.

**Figure 2:**
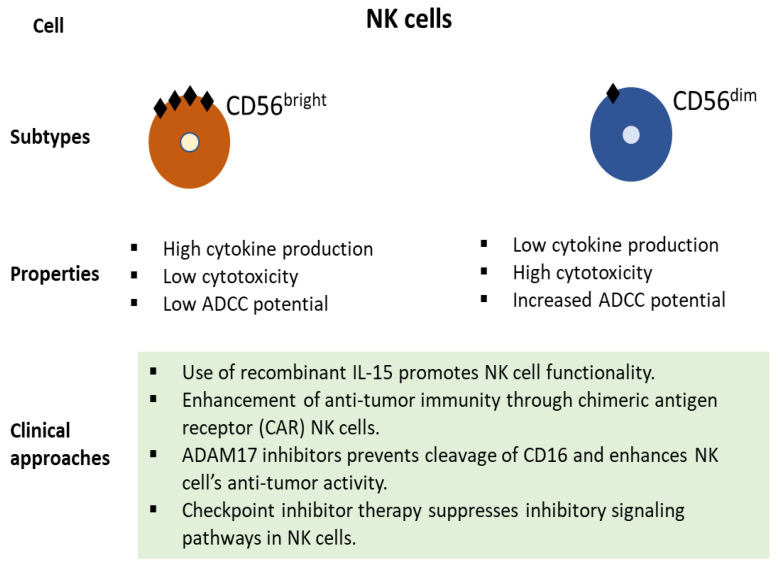
Broad subtypes of NK cells, their function, and potential clinical approaches with therapeutic benefits.

Recent advances in cancer therapies have led to the emergence of ICB with promising benefits in some segments of patients. However, immune-mediated adverse effects, tumor heterogeneity, high costs, and complexity have brought unique challenges to its expansion to the wider population [93]. ICB has an objective response rate (ORR) in only 26% of patients across all types of cancer [94]. The patient’s subset that is resistant to ICB and low TMB can benefit from NK cell-based therapies [95,96]. Interestingly, higher infiltration of NK cells was found to be associated with response to anti-PD-1 therapy [97]. NK cells might play an important role in the tumor subsets that exhibit loss of neoantigen presentation due to downregulation of MHC-I molecules [98]. Additionally, NK cell-DC axis may provide a prognostic tool for immunotherapy as it enhances T cell response [88]. In NSCLC, NK cell gene signature was found to be associated with the anti-PD1 treatment response and PFS [99]. In another study of advanced NSCLC patients, the pool of CD8+ T cells and NK cells predicted the outcome of anti-PD1 therapy [100]. The clinical use of NK cells can be exploited through several strategies (Figure 2). The functionality of NK cells can be enhanced using recombinant IL-15 or chimeric antigen receptor (CAR) NK cells [101,102]. Further, the immune cytotoxicity of NK cells can be enhanced using ADAM17 (A disintegrin and metalloprotease 17) inhibitors [103]. The inhibition of ADAM17 leads to strengthened tethering of antibodies, reduced detachment of tumor cells, and increased production of cytokines [103,104]. Furthermore, the inhibitory signals of NK cells are suppressed by checkpoint inhibitor therapy [105]. In NSCLC, several advances have been made in understanding the prognostic significance of NK cells (Table 1). Circulating NK cells were found to be prognostically significant in NSCLC patients [106]. Moreover, the number of NK cells reduces in number in peripheral blood after chemotherapy [107]. In quantifying the response to ICB therapies, the circulating pool of NK cells was found to be predictive of beneficial response in NSCLC patients [100]. These recent findings will help in expanding the prognostic and predictive role of NK cells in NSCLC patients.

## 3. The Emerging Role of Dendritic Cells in the Tumor Microenvironment and Immunotherapy

DCs (Dendric cells) play an important role in the initiation, development, and maintenance of the anti-tumor immune response. Dying cells release damage-associated molecular patterns (DAMPs), which are predominantly intracellular proteins and induce the production of cytokines and activation of T cells [113]. Upon uptake of antigens, DCs undergo maturation and migrate to lymph nodes, where they present the antigens to CD8+ T cells [114]. DCs are broadly divided into two classes, conventional type 1 DCs (cDC1s) and conventional type 2 DCs (cDC2s). cDC1s functions primarily by cross-presenting antigens to CD8+ T cells. In contrast, cDC2s function by priming CD4+ T cell response [115]. Furthermore, based on transcriptional and chromatin variations, cDC2s have been divided into anti-inflammatory cDC2A (T-bet^+^) and pro-inflammatory cDC2B (T-bet^-^) [116]. cDC1s are essential for mounting an anti-tumor immune response. cDC1s infiltration has been found to positively correlate with T cell infiltration and increased survival. Tumor evasi strategies include the prevention of cDC1 infiltration into the tumor microenvironment [87]. cDC1 binds to F-actin exposed necrotic bodies through C-type lectin receptor DNGR-1. This process leads to the uptake and cross-presentation of antigens from dead cell debris to initiate CD8+ T cell response [117]. Recently, tumor-secreted gelsolin (sGSN) has been found to dampen the immune response by impairing the DNGR-1-dependent cross-presentation in cDC1 [118]. The third class of DCs is known as plasmacytoid dendritic cells (pDC), with appearances like plasma cells and characteristic production of high levels of interferon-α [119]. Tumor-promoting features of aberrant pDCs with poor production of type-I IFN and T-reg differentiation were displayed in patients with breast and ovarian cancer [120,121]. The molecular basis of the differentiation of DCs in the tumor microenvironment is an active area of research and may hold promise in the development of future immunotherapies.

Increased infiltration, expansion, and activation of cDC1s play an important role in the immune control of tumors and response to immunotherapies [87,122]. Interestingly, the DC gene signature was found to be associated with improved overall survival in NSCLC patients treated with tezolizumab (PD-L1 blockade) [123]. There are two pre-dominant receptors for PDL-1: PD-1 and B7.1. In an interesting study, the expression of PDL-1 was found to be significantly higher in DCs present in TME and circulation of cancer patients. The blockage of PD-L1 relieved B7.1 that in turn interacted with CD28 to enhance the priming of T cells [123]. Additionally, dendritic cells are also essential in the reactivation of circulating memory T cells [124]. In another study, anti-PD-1 immunotherapy was found to depend on IL-12-secreting DCs in the presence of IFN-γ-secreting T cells [125]. Several factors prevent the anti-tumor effect of DCs in the complex microenvironment of the tumor. The recruitment of cDCs is sparse in the tumor microenvironment in early-stage tumors compared to adjacent normal tissue. The presence of NK cells was found to be significantly reduced in lung adenocarcinoma patients.NK cells in these tumors showed poor cytolytic capacity due to the lower expression of granzyme B, CD57, and IFN-γ [126]. This mechanism could in part be responsible for preventing the anti-tumor immune response [126]. Furthermore, the WNT/β-catenin pathway in tumors can partly prevent the infiltration of cDCs and T cells. Activation of this pathway in the tumor impedes the expression of CCL4, which reduces infiltration of DCs in the tumor. The resulting reduced CXCL10 limits CD8+ T cells and leads to faulty cross-priming [127]. Moreover, the presence of prostanoid lipids leads to the expansion of tumor growth, migration, invasion, and immunosuppression [128]. Necrosis in the tumor releases prostaglandin E2 (PGE_2_) and it has been shown to prevent the immunostimulatory activity of DCs [129]. The overexpression of COX1 and 2 Cyclooxygenase (COX) and production of PGE_2_ in the hypoxic microenvironment prevent the accumulation and activation of cDCs and assists in immune evasion [130,131]. The presence of Vascular endothelial growth factor (VEGF) in the tumor microenvironment is also a suppressing factor of DCs, as it adversely affects functionality [132,133]. Recently, a new subset of DCs, ‘mature DCs, enriched in immunoregulatory molecules’ (mregDCs), with an immunoregulatory gene signature (*Cd274*, *Cd200*, and *Pdcd1lg2*), has been identified [134]. These cDCs continue with the uptake of antigens, but do not stimulate T cell activation in lymph nodes, blocking the trajectory of inflammation. DCs with similar regulatory gene signatures have been identified in normal tissue, which hints at its role in the maintenance of homeostasis. Furthermore, the process of cross-presentation can itself be impaired in the tumor tissue due to the lack of tumor-infiltrating DCs with activating potential [134,135]. Increased production of oxidized lipids in DC adversely affects the cross-presentation process [136,137,138]. One of the reasons for this could be the increased uptake of lipids due to higher expression of scavenging receptor MSR1 in DCs [136]. In another study, infiltrated DCs exhibited ER stress and expression of ER stress response factor XBP1 promoted primary and metastatic ovarian cancer [137]. It has also been reported that DCs with increased lipid content failed to effectively present antigens or stimulate T cells. It was found that oxidized lipids sequester chaperone HSP70; thus, preventing the MHC-peptide complex from reaching the cell surface [138].

Despite these challenges, there are promising approaches that could assist in the expansion of DCs as a central player in future therapeutic strategies. In pre-clinical models, poly I:C (TLR-3, MDA5, and RIG-I. agonist) treatment led to increased IFNα/β-related transcriptomic profile, and increased infiltration of dendritic cells and T cell in the melanoma mouse model [139]. The intra-tumoral activation of STING pathway was found to initiate an immune response and led to regression of established tumors. It was found that STING agonists led to the maturation of DCs and the production of cytokines and chemokines [140]. Modified amidobenzimidazole (ABZI)-based compounds have also been developed to enhance the STING pathway [141]. Also, VEGF blockade therapy through anti-VEGF antibody has been shown to stimulate DCs and T cells to enhance tumor immunity [142]. Infiltration of DCs led to an improved response to checkpoint inhibitor immunotherapy and the administration of tumor-stroma-directed CCL4 administration through the intravenous method led to increased infiltration of DCs and CD8+ T cells even in poor responders to checkpoint inhibitor (CPI) immunotherapy [143]. cDC1 abundance has been reported to be associated with checkpoint blockade immunotherapy [88,144]. Overall, the composition of the TME and infiltrating immune cells play a critical role in determining the efficacy of Checkpoint inhibitors [145]. There is significant interest in the identification of the immune cells and their associated variables that determine the responsiveness to immunotherapy.

**Figure 3:**
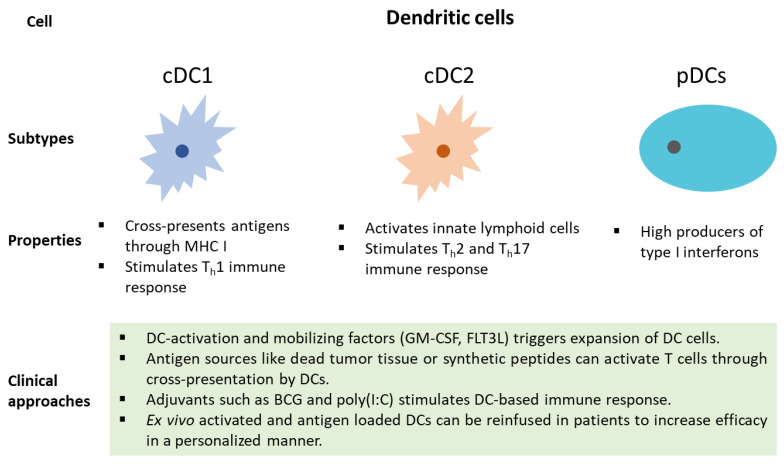
Types of DCs, their function, and their clinical approaches to tap into their potential for the therapeutic benefit of cancer patients.

Many clinical approaches can be utilized to harness the potential of DCs in cancer patients (Figure 3). Dendritic cell mobilizing factors, such as GM-CSF and FLT3L, can lead to the expansion of DC cells [146]. Additionally, DC-mediated T cell activation through the presentation of antigens and synthetic peptides can lead to an anti-tumor immune response [147]. Furthermore, DC response can be stimulated using adjuvants such as BCG and poly(I:C) [148]. In a personalized approach, ex vivo activated and antigen-loaded DCs can be reinfused in cancer patients [147]. Recently, DCs pulsed with survivin and MUC1 showed promise in resected NSCLC [149]. Advances in the prognostic roles of DCs in NSCLC have hinted at its critical role in the TME (Table 2). DCs are positively correlated with the progression-free survival of NSCLC patients [150]. The expression of individual genes, such as TOP2A and TLR3, and multiple gene signatures have been associated with DC infiltration in NSCLC [151,152,153]. These recent findings related to gene expression signatures and infiltration of DCs in NSCLC will assist in the design of effective strategies for patients with refractory cancer.

## 4. NK Cells and DCs: Prospects in NSCLC

In NSCLC, treatment strategies involved in the management of cancer patients range from conventional chemotherapy regimens to newly approved immunotherapeutic agents [158]. Despite its recent success, several challenges associated with immunotherapy include lack of consistent response, lack of predictive biomarkers, risk of immune-related adverse effects, and resistance to immunotherapy [159]. NK cells and DCs have started to emerge as critical players in the development of a newer generation of therapeutic strategies. For example, monoclonal antibodies targeting NK inhibitory receptors and IL-15 can activate killer properties of NK cells [160]. In a recent clinical trial, patients administered with Pembrolizumab plus NK cell therapy showed improved survival in advanced NSCLC cases. In these patients, infusion of NK cells led to increased circulation of NK cells in the blood and enhancement of cellular immune functions. Interestingly, this combinatorial therapy also led to a reduction in circulating tumor cells [161]. The role of NK cells as a promising therapeutic modality is actively being investigated. Another strategy explored the clinical potential of DC cells as a vaccine in NSCLC patients. In this trial, DCs were silenced for SOCS1 expression (to prevent negative regulation of DCs), pulsed with survivin and MUC1 (heavily expressed proteins in NSCLC tumor), and flagellin (immune stimulant) [149,162]. It was shown that the administration of DCs vaccine led to a reduction in tumor markers and improved the quality of life in cancer patients [149]. In another trial, the efficacy of the adenoviral vector with the CCL21 gene (Ad-CCL21-DC) was explored in stage III/IV NSCLC patients [163]. Higher expression of CCL21 attracts T cells and DCs through interaction with CXCR3 CCR7 receptors [163,164]. In mouse models, CCL21 treatment led to higher infiltration of DCs CD4+ and CD8+ in tumor [165]. Administration of intra-tumoral vaccination led to enhanced infiltration of CD8+ T cell, increased expression of PD-L1 in tumors, and induction of immune response against tumor antigen [163]. In lung tumors, higher infiltration of mature dendritic cells was correlated with the influx of effector T cells [166]. This study identified the presence of tertiary lymphoid structures enriched in DCs, T_h_1 subtype and cytotoxic properties with improved prognostic outcomes in the tumor microenvironment [166]. In addition to the therapeutic benefits of NK and DCs, gene signatures associated with the infiltration of these cells can provide vital prognostic and predictive biomarkers [106,110,150,153,157]. Encouragingly, the preliminary results of clinical trials involving NKs and DCs are most likely to play a key role in the design and application of future immunotherapies.

## 5. Conclusions

Globally, lung cancer has one of the highest cases of all cancers. In addition to the therapeutic benefits of immunotherapy, strategies involving DCs and NK cells have started to emerge as promising candidates to combat this disease. DCs play a critical role in the control of tumor progression but their function is limited due to the immunosuppressive microenvironment of the tumor. Recent strategies to activate DC-based immunity through DC vaccines and the modification of TME have appeared to be a promising approach to prevent tumor growth. One of the major barriers to exploiting DC-based immune control strategies is the immunosuppressive microenvironment. Novel strategies to understand and utilize the critical mediators of DCs function would be central for its benefits to reach cancer patients. Similarly, the durability of NK cell-based approaches suffers a bottleneck due to the lack of tumor-specific NK cells, low TMB, and ineffective activation. To maximize the clinical benefits, immunotherapies based on DCs and NK cells can be combined with other therapies, such as chemotherapy, radiotherapy, and ICB. Furthermore, continued research is critical to identify prognostic and predictive markers associated with individual immunotherapies, their combinations and will likely play a critical role in the development of multimodal strategies to combat cancer.

## Figures and Tables

**Table 1:** Recent advances in identifying prognostic roles of NK cells in NSCLC.

S. No.	The Theme of the Study	Clinical Significance	Reference
1	A new approach to isolate NK cell-derived exosomes for therapeutic benefits in NSCLC	A highly sensitive and specific technique to isolate and identify cytotoxic exosomes from NK cells.	[108]
2	The role of NK-mediated protection against ROS in TME	NK cells in the tumor microenvironment expres a higher concentration of thiols, and can prevent oxidative damage to other lymphocytes.	[109]
3	Inhibitory profile of NK cells in NSCLC	Transcriptome analysis of NK cells identified downregulation of S1PR1 and CX3CR1 and upregulation of CXCR5 and CXCR6 along with increased expression of CTLA-4 and KLRC1.	[110]
4	Peripheral NK cells and chemotherapy in NSCLC	The level of NK cells showed a decline after chemotherapy in peripheral blood of NSCLC patients.	[107]
5	Prognostic significance of NK cells in circulation	Circulating NKp46+ CD56dim CD16+ NK cells were prognostically significant in NSCLC patients.	[106]
6	Natural killer cell activity assay	Low Natural killer cell activity (NKA) was found to be a clinical signature of NSCLC.	[111]
7	NK cells and ICB response	The circulating pool size of CD8+ T cells and NK cells were found to be predictive of ICB response.	[100]
8	NK cell as a biomarker from blood	The quantification of NK cell receptors from blood samples can be used to stratify patients.	[112]

**Table 2:** Recent advances in identifying prognostic roles of DCs cells in NSCLC.

S. No.	The Theme of the Study	Clinical Significance	Reference
1	Autologous modified DC vaccine	DC vaccine pulsed with MUC1, survivin, and flagellin was well tolerated and induced anti-tumor activity.	[149]
2	Prognostic significance of pDCs and mDCs	There was a significant reduction of plasmacytoid dendritic cells (pDCs) and monocytic dendritic cells (mDCs) in NSCLC patients. The overall survival was negatively correlated to mDCs but positively correlated with pDCs.	[154]
3	Prognostic association of DCs	DCs showed significant association with PFS and disease stage in NSCLC patients.	[150]
4	MYEOV gene expression was found to be prognostically significant	The expression of the myeloma overexpressed gene (MYEOV) was found to be associated with poorer overall survival and increased infiltration of immune cells, including DCs.	[155]
5	TP53 mutations were associated with poor survival	There was higher infiltration of immune cells, including CD8+ T cells and DCs in TP53-mutated lung cancer tissues.	[156]
6	TLR3 expression was prognostically significant in NSCLC patients	The presence of the TLR3-CD1-3+ Dendritic cell axis and corresponding activation of CD8+ T cells was found to be associated with improved overall survival.	[151]
7	5-gene prognostic signature	There was higher infiltration of DCs in the lower risk group compared to a higher risk group.	[153]
8	Identification of differentially expressed genes	TOP2A expression was found to be significantly associated with the infiltration of DCs.	[152]
9	Association of DCs with anti-tumor immunity	Patients with the non-metastatic disease had higher infiltration of dendritic cells.	[157]
